# Association between preoperative neutrophil percentage-to-albumin ratio and survival outcomes in patients with colorectal cancer undergoing radical surgery: a retrospective study with external validation

**DOI:** 10.3389/fonc.2026.1775482

**Published:** 2026-07-21

**Authors:** Alafate Yilamu, Aikeremu Yusufu, Saibihutula Ababek, Xiakejiang Rexiti

**Affiliations:** 1 The Third Clinical Medical College of Xinjiang Medical University, Urumqi, Xinjiang, China; 2 Department of Gastrointestinal Surgery (Ward 2), Affiliated Cancer Hospital of Xinjiang Medical University, Urumqi, Xinjiang, China

**Keywords:** colorectal cancer, neutrophil percentage-to-albumin ratio (NPAR), preoperative assessment, prognosis, systemic inflammation

## Abstract

**Background:**

Accurate preoperative risk stratification remains a major clinical challenge in patients with colorectal cancer (CRC) undergoing radical surgery. Simple and readily available biomarkers that reflect systemic inflammation and nutritional status may improve prognostic assessment in routine clinical practice.

**Methods:**

This retrospective study included 581 patients with CRC who underwent radical surgery as the training cohort. Overall survival (OS) and disease-free survival (DFS) were the primary endpoints. NPAR was analyzed primarily as a continuous variable standardized per 1-SD increase. A pre-specified final model including NPAR, age, sex, tumor site, pathological stage, and log10-transformed CEA was used consistently across Cox regression, nomogram development, time-dependent ROC analysis, calibration, decision-curve analysis, and external validation. Dichotomized NPAR was retained only for descriptive and supplementary analyses. External validation was performed in 483 patients from an external clinical database.

**Results:**

In the training cohort, higher preoperative NPAR was associated with adverse clinicopathological features. After adjustment for age, sex, tumor site, pathological stage, and log10-transformed CEA, each 1-SD increase in NPAR was associated with shorter OS (HR 2.19, 95% CI 1.90–2.53, *p* < 0.001) and DFS (HR 1.88, 95% CI 1.62–2.19, *p* < 0.001). The final model showed optimism-corrected C-indexes of 0.835 for OS and 0.752 for DFS in the training cohort. In the external cohort, the association between continuous NPAR and outcomes was directionally consistent but attenuated for both OS (HR 1.21, 95% CI 1.08–1.35, *p* < 0.001) and DFS (HR 1.24, 95% CI 1.12–1.37, *p* < 0.001). External C-indexes were 0.690 for OS and 0.705 for DFS. Adding NPAR to the baseline clinicopathological model did not improve external time-dependent discrimination for OS and showed only limited incremental value for DFS. Low external recalibration slopes suggested possible optimism in the training model.

**Conclusion:**

Preoperative NPAR showed an adjusted association with OS and DFS, and the direction of this association was reproduced in the external cohort. However, its incremental predictive value beyond conventional clinicopathological factors was limited, particularly for OS. NPAR may be considered a simple adjunctive marker of inflammatory and nutritional status, but its clinical utility for individualized risk prediction requires further validation in larger prospective cohorts.

## 1 Introduction

Colorectal cancer (CRC) is one of the most common malignant tumors of the digestive system and remains a major cause of cancer-related death worldwide. According to the International Agency for Research on Cancer (IARC) in 2022 (1), more than 1.9 million new CRC cases and approximately 904,000 deaths were recorded globally. CRC ranks among the leading malignancies in both incidence and mortality worldwide (2). National epidemiological studies in China (3, 4) have likewise shown that CRC remains a major public health burden, with both incidence and mortality continuing to rise. Histopathologically, adenocarcinoma is the predominant subtype of CRC and accounts for more than 90% of all cases (5).

At present, radical surgery is considered the fundamental approach for treating colorectal cancer (CRC). Nonetheless, persistently high rates of postoperative recurrence and metastasis have complicated efforts to markedly enhance long-term survival rates. In this regard, improving the accuracy of preoperative assessments for patients at high risk is crucial for developing personalized treatment plans, optimizing follow-up management approaches, and enhancing clinical outcomes. While the current AJCC 8th edition TNM staging system (6) is a valuable resource for clinical risk assessment, it primarily emphasizes tumor morphological features and does not sufficiently consider the variations in systemic inflammatory responses, nutritional metabolism, and immune system activity. This oversight results in substantial prognostic differences among patients categorized within the same staging group, underscoring the limitations of the TNM system for personalized prognostic evaluation. Recent studies indicate that the systemic inflammatory response is vital in driving tumor progression (7–9). Numerous investigations have validated the potential significance of inflammatory markers in peripheral blood, such as the neutrophil-to-lymphocyte ratio (NLR), platelet-to-lymphocyte ratio (PLR), and lymphocyte-to-monocyte ratio (LMR), for prognostic evaluation in various types of cancer (10–12). Nevertheless, it is important to recognize that these markers only indicate specific elements of inflammatory responses and do not provide a complete assessment of a patient’s nutritional and metabolic condition. Conversely, serum albumin serves as a multifaceted indicator that reflects nutritional reserves, inflammatory status, and liver functionality, with its lower levels often correlated with malnutrition, worsened inflammatory conditions, and a higher likelihood of postoperative complications (13, 14). Consequently, developing a comprehensive evaluation system that combines both inflammatory and nutritional statuses is anticipated to facilitate a more accurate overall assessment of the patient’s health.

Among inflammation- and nutrition-related biomarkers, the neutrophil percentage-to-albumin ratio (NPAR) has recently attracted increasing attention. NPAR integrates two clinically relevant dimensions of cancer progression, namely systemic inflammatory activation and impaired nutritional status, and has shown prognostic value in several malignancies (15–17). Emerging evidence has also suggested that NPAR may be associated with survival outcomes in colorectal cancer (18, 19). However, the currently available evidence in CRC remains limited by heterogeneity in study populations, cutoff determination, endpoint definitions, and the lack of external validation. In addition, whether NPAR provides incremental prognostic information beyond conventional clinicopathological factors and other routinely available inflammatory markers remains insufficiently clarified.

Therefore, the aim of the present study was to evaluate the prognostic relevance of preoperative NPAR in patients with colorectal cancer undergoing radical resection, to develop NPAR-integrated prognostic models for OS and DFS, and to assess their transportability in an external validation cohort. Compared with previous CRC studies, the present study additionally incorporated detailed clinicopathological characterization, dynamic model evaluation using time-dependent ROC, calibration and decision curve analyses, and external validation.

## 2 Materials and methods

### 2.1 Study design and training cohort

This retrospective cohort study was conducted at the Affiliated Cancer Hospital of Xinjiang Medical University. The training cohort included consecutive patients who underwent radical surgery for colorectal cancer in the Department of Gastrointestinal Surgery between 2017 and 2019. The inclusion criteria were as follows: (1) age >18 years; (2) histopathologically confirmed colorectal adenocarcinoma after surgery; (3) first-time radical resection for colorectal cancer; and (4) relatively complete preoperative clinical data, laboratory test results, and follow-up information. The exclusion criteria were as follows: (1) non-radical resection or prognosis that could not be adequately evaluated (n= 4); (2) death during the perioperative period (n= 2); (3) receipt of neoadjuvant chemoradiotherapy before surgery (n = 47); (4) definite active infection within 1 month before surgery or other acute conditions that could substantially affect inflammatory or nutritional indices (n = 3); and (5) missing key clinical data, laboratory results, or follow-up information that could not be completed (n = 9). Ultimately, 581 patients were included in the training cohort.

#### 2.1.1 External validation cohort

**Figure 1:**
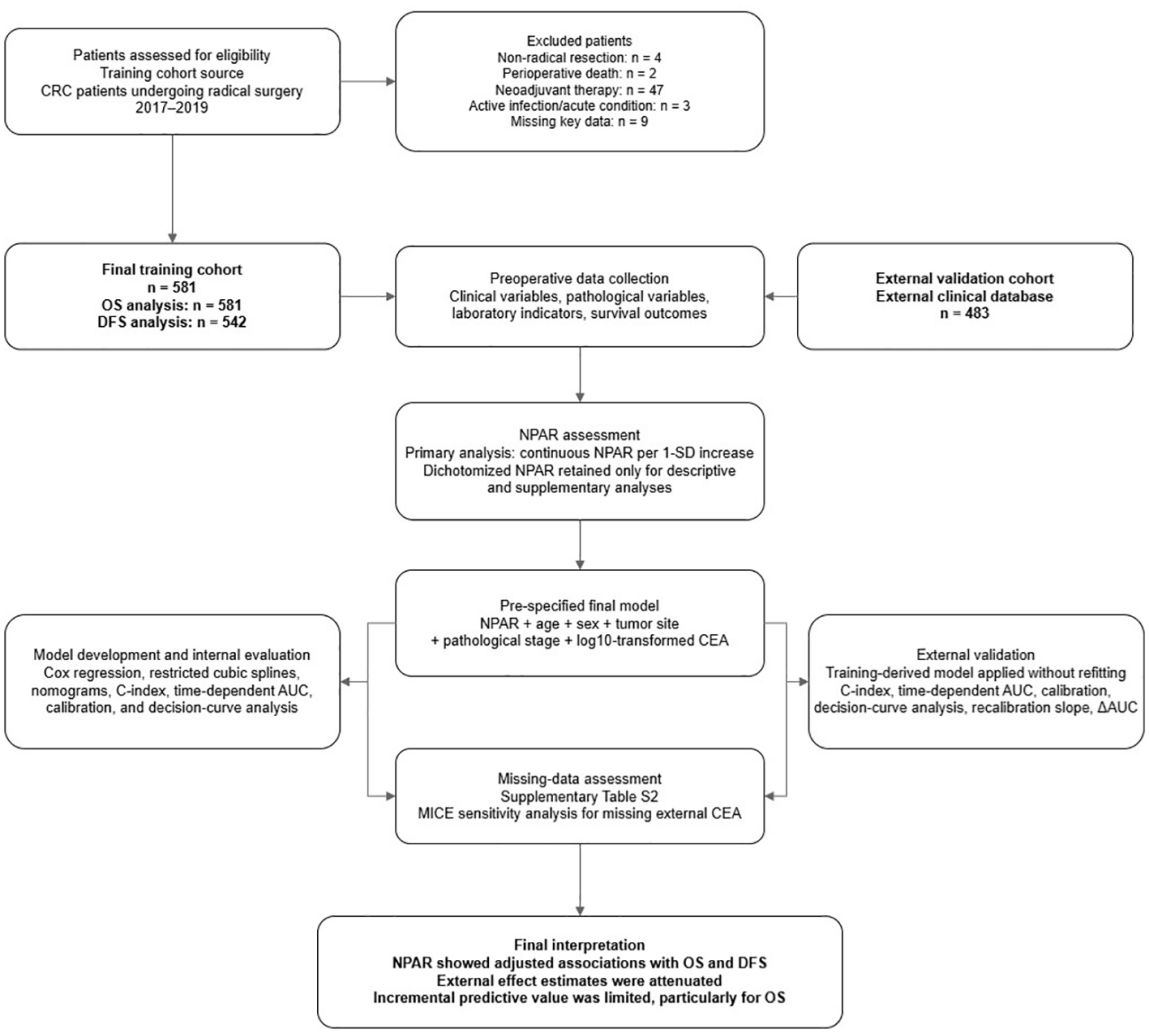
Integrated study workflow for cohort screening, preoperative NPAR assessment, prognostic model development, internal performance evaluation, missing-data assessment, and external validation.

The external validation cohort was obtained from a publicly available dataset hosted on the Science Data Bank (CSTR:31253.11.sciencedb.j00253.01901), which contains clinical, laboratory, imaging, and follow-up data of patients with colorectal cancer from the First Affiliated Hospital of Kunming Medical University (20). Because the public record did not provide a fully reconstructable original screening flow or detailed exclusion counts, the original source population could not be precisely determined. Therefore, in the present study, all cases with available preoperative variables required for NPAR calculation and with evaluable survival outcome data were included in the external validation analysis. This cohort comprised 483 analyzable patients and was used to assess the external performance of the NPAR-based model. The overall study workflow, including cohort screening, preoperative NPAR assessment, prognostic model development, internal performance evaluation, missing-data assessment, and external validation, is summarized in **Figure 1**.

### 2.2 Data collection

All laboratory assays were performed in the central clinical laboratory of the Affiliated Cancer Hospital of Xinjiang Medical University according to standardized operating procedures. Peripheral venous blood samples were collected within two weeks before surgery in accordance with institutional preoperative testing protocols. Hematological parameters, including neutrophil, lymphocyte, monocyte, and platelet counts, were measured using the Sysmex XN-9000 hematology analyzer, whereas serum albumin was measured using the Roche Cobas C701 automated biochemical analyzer. All assays were performed by trained laboratory personnel. The laboratory implemented routine internal quality control and participated in regular external quality assessment programs, and the analyzers underwent periodic calibration and maintenance in accordance with the manufacturers’ instructions. To reduce preanalytical variability, only preoperative blood test results obtained within two weeks before surgery were included in the analysis. Following the literature and the study’s criteria, various inflammatory and nutritional indicators were computed as follows:


$$NPAR=\frac{Neutrophil\;percentage(\%)\times 100}{\;\mathrm{Albumin}(g/L)},$$

NLR = Neutrophil count/lymphocyte count, PLR = Platelet count/lymphocyte count, LMR = Lymphocyte count/monocyte count.

### 2.3 Follow-up and survival endpoints

Follow-up was initiated on the date of surgery and continued until December 31, 2023, or death, whichever occurred first. According to institutional follow-up practice, patients were generally followed every 3 months during the first 2 postoperative years, every 6 months during years 3 to 5, and annually thereafter. Follow-up information was obtained through outpatient visits, telephone interviews, review of imaging records, and medical record documentation. The study endpoints were defined as follows: overall survival (OS), calculated from the date of surgery to death from any cause or the date of last follow-up; and disease-free survival (DFS), calculated from the date of surgery to the first documented recurrence, distant metastasis, death, or the date of last follow-up, whichever occurred first. Recurrence or distant metastasis was confirmed either by pathological evidence or by imaging findings highly suggestive of recurrence or metastasis on CT, MRI, PET-CT, or endoscopy. Because recurrence-related follow-up information was incomplete in a subset of patients, the effective sample size for DFS analysis was smaller than that for OS analysis. Detailed information on sample selection and sample size for each analysis is provided in the Results section.

### 2.4 Statistical analysis

All statistical analyses were performed using R software, version 4.3.2. The main packages used included survival, rms, timeROC, mice, Hmisc, survminer, and survIDINRI. All statistical tests were two-sided, and a *p* value <0.05 was considered statistically significant. P values were reported to three decimal places, with values <0.001 presented as *p* < 0.001. Continuous variables were summarized as median and interquartile range (IQR), because the Shapiro–Wilk test indicated that most continuous variables were not normally distributed. Between-group comparisons were performed using the Wilcoxon rank-sum test. Categorical variables were summarized as frequencies and percentages and compared using the chi-square test or Fisher’s exact test, as appropriate. NPAR was analyzed primarily as a continuous variable. To improve comparability between cohorts and to facilitate interpretation, NPAR was standardized according to the mean and standard deviation of the training cohort, and hazard ratios were reported per 1-SD increase. A data-derived cutoff value of 1.55, identified in the training cohort using the ROC curve and Youden index, was retained only for descriptive grouping and supplementary analyses. Because dichotomization of continuous variables may lead to information loss and overestimation of effect sizes, all primary Cox regression, nomogram, discrimination, calibration, and external validation analyses were based on continuous NPAR. The association between NPAR and survival outcomes was evaluated using Cox proportional hazards models. Overall survival (OS) and disease-free survival (DFS) were analyzed separately. A single pre-specified final model was used consistently across all main analyses. This model included continuous NPAR per 1-SD increase, age, sex, tumor site, pathological stage categorized as I–II versus III–IV, and CEA. Because CEA showed a skewed distribution, it was transformed using log10(x+1) before model fitting. To reduce multicollinearity, pathological stage was used instead of simultaneously entering pT, pN, and pM categories, and tumor site was used without simultaneously entering detailed tumor location. Collinearity was assessed using variance inflation factors and Spearman correlation coefficients among continuous predictors. Potential nonlinearity in the association between continuous NPAR and survival outcomes was examined using restricted cubic splines. The proportional-hazards assumption was assessed using Schoenfeld residuals. When evidence of non-proportional hazards was observed, the corresponding hazard ratio was interpreted as an average association over the follow-up period rather than as a time-constant effect. Kaplan–Meier curves were generated for descriptive visualization of survival differences across NPAR tertiles and compared using the log-rank test. The dichotomized NPAR cutoff was used only in supplementary Kaplan–Meier and Cox analyses. Model discrimination was evaluated using Harrell’s C-index and time-dependent area under the ROC curve at clinically relevant time points. Internal optimism in the training cohort was estimated using bootstrap resampling with 1000 iterations. Nomograms were constructed from the final Cox model. Calibration was assessed using bootstrap calibration plots. Clinical utility was explored using decision-curve analysis. External validation was performed in an independent cohort by applying the final model derived from the training cohort without re-estimating model coefficients. External performance was assessed using Harrell’s C-index, time-dependent AUC, calibration plots, decision-curve analysis, and recalibration slope. The recalibration slope was estimated by fitting a Cox model with the training-derived linear predictor as the only covariate in the external cohort. A slope close to 1 was considered to indicate better transportability, whereas a low slope suggested potential overfitting or optimism in the training model. The incremental value of NPAR was evaluated by comparing the final model with a baseline model that included the same clinicopathological variables but excluded NPAR. Changes in time-dependent AUC were calculated at predefined time points in the external cohort. Bootstrap resampling with 1000 iterations was used to estimate 95% confidence intervals for changes in AUC. Missingness was summarized for all variables included in the final model and for outcome-specific follow-up variables. Outcome variables were not imputed. OS analyses included patients with evaluable OS, whereas DFS analyses included patients with evaluable recurrence or metastasis follow-up. For covariates included in the final model, complete-case analysis was used as the primary approach. Because CEA was missing in 60 patients in the external cohort, multiple imputation by chained equations with 20 imputations was performed as a sensitivity analysis. Results from complete-case and imputed analyses were compared to assess the robustness of the external validation findings.

### 2.5 Ethical statement

This study was approved by the Institutional Ethics Committee of our hospital (approval number:K-2025158) and was conducted as a retrospective study, with a waiver of written informed consent. All patient information was anonymized in strict compliance with the ethical principles of the Declaration of Helsinki.

## 3 Results

### 3.1 Baseline characteristics of the study population

A total of 581 patients with colorectal cancer were included in the training cohort (**Table 1**). The median follow-up duration was 57 months [interquartile range (IQR), 43–67 months]. Of the 581 patients, 352 (60.6%) were male and 229 (39.4%) were female. The primary tumor was located in the rectum in 310 patients (53.4%) and in the colon in 271 patients (46.6%). Moderately differentiated tumors were the most common histological subtype (n = 497, 85.5%). According to pathological stage, 96 patients (16.5%) had stage I disease, 206 (35.5%) had stage II disease, 225 (38.7%) had stage III disease, and 54 (9.3%) had stage IV disease. Most patients had deep tumor invasion (pT3–4, 79.5%), 263 (45.3%) had regional lymph node metastasis (pN1–2), and 54 (9.3%) had distant metastasis (pM1), indicating that the cohort mainly consisted of patients with intermediate-to-advanced disease. For survival analyses, all 581 patients were included in the OS analysis. For DFS analysis, the effective sample size was 542 because follow-up information on recurrence or metastasis was incomplete in a subset of patients. Accordingly, **Table 1** summarizes the baseline characteristics of the overall training cohort, whereas **Tables 2** and **3** present the Cox regression analyses for OS and DFS, respectively. Missing data are summarized in **Supplementary Table 2**. In the training cohort, variables included in the final OS model were complete, and all 581 patients were included in the OS analysis. DFS follow-up information was unavailable in 39 patients (6.7%), resulting in 542 evaluable patients for DFS analysis. In the external cohort, age, sex, tumor site, pathological stage, NPAR, OS, and DFS variables were complete, whereas CEA was missing in 60 patients (12.4%). Multiple imputation was therefore used as a sensitivity analysis for external validation.

**Table 1:** Baseline clinicopathological characteristics of the training cohort stratified by preoperative NPAR.

Variable	Overall	Low NPAR group (NPAR<1.55, n = 358, 61.6%)	High NPAR group (NPAR≥1.55, n = 223, 38.4%)	*p* value
Sex				0.030
Male	352 (60.6%)	204 (57.0%)	148 (66.4%)	
Female	229 (39.4%)	154 (43.0%)	75 (33.6%)	
Age				<0.001
<68.50	401 (69.0%)	267 (74.6%)	134 (60.1%)	
≥68.50	180 (31.0%)	91 (25.4%)	89 (39.9%)	
Tumor site				0.800
Colon	271 (46.6%)	165 (46.1%)	106 (47.5%)	
Rectum	310 (53.4%)	193 (53.9%)	117 (52.5%)	
Tumor location				0.232
Transverse colon	19 (3.3%)	11 (3.1%)	8 (3.6%)	
Right colon	72 (12.4%)	37 (10.3%)	35 (15.7%)	
Rectum	312 (53.7%)	194 (54.2%)	118 (52.9%)	
Descending colon	178 (30.6%)	116 (32.4%)	62 (27.8%)	
Differentiation				0.217
Poorly	62 (10.7%)	33 (9.2%)	29 (13.0%)	
Moderately	497 (85.5%)	309 (86.3%)	188 (84.3%)	
Well	22 (3.8%)	16 (4.5%)	6 (2.7%)	
Pathological stage				<0.001
I	96 (16.5%)	70 (19.6%)	26 (11.7%)	
II	206 (35.5%)	144 (40.2%)	62 (27.8%)	
III	225 (38.7%)	132 (36.9%)	93 (41.7%)	
IV	54 (9.3%)	12 (3.4%)	42 (18.8%)	
pT				0.018
pT1-2	119 (20.5%)	85 (23.7%)	34 (15.2%)	
pT3-4	462 (79.5%)	273 (76.3%)	189 (84.8%)	
pN				<0.001
pN0	318 (54.7%)	219 (61.2%)	99 (44.4%)	
pN1-2	263 (45.3%)	139 (38.8%)	124 (55.6%)	
pM				<0.001
pM0	527 (90.7%)	346 (96.6%)	181 (81.2%)	
pM1	54 (9.3%)	12 (3.4%)	42 (18.8%)	
BMI				0.074
<24.04	253 (43.5%)	145 (40.5%)	108 (48.4%)	
≥24.04	328 (56.5%)	213 (59.5%)	115 (51.6%)	
Tumor size				<0.001
<4.25 cm	266 (45.8%)	184 (51.4%)	82 (36.8%)	
≥4.25 cm	315 (54.2%)	174 (48.6%)	141 (63.2%)	
CEA				0.007
Negative	367 (63.2%)	242 (67.6%)	125 (56.1%)	
Positive	214 (36.8%)	116 (32.4%)	98 (43.9%)	
CA19-9				0.088
Negative	506 (87.1%)	319 (89.1%)	187 (83.9%)	
Positive	75 (12.9%)	39 (10.9%)	36 (16.1%)	
CA125				<0.001
Negative	552 (95.0%)	351 (98.0%)	201 (90.1%)	
Positive	29 (5.0%)	7 (2.0%)	22 (9.9%)	
MMR status				0.911
pMMR	545 (93.8%)	335 (93.6%)	210 (94.2%)	
dMMR	36 (6.2%)	23 (6.4%)	13 (5.8%)	
Absolute lymphocyte count (10^9^/L), median (IQR)	1.73 (1.35–2.12)	1.87 (1.49–2.21)	1.48 (1.17–1.91)	<0.001
Absolute neutrophil count (10^9^/L), median (IQR)	3.57 (2.85–4.41)	3.15 (2.54–3.89)	4.27 (3.52–5.44)	<0.001
Absolute monocyte count (10^9^/L), median (IQR)	0.43 (0.34–0.55)	0.41 (0.33–0.52)	0.46 (0.37–0.60)	<0.001
Albumin (g/L), median (IQR)	41.00 (37.90–43.50)	41.90 (39.70–44.40)	38.50 (36.10–41.40)	<0.001

Categorical variables were compared using the chi-square test or Fisher’s exact test, as appropriate. Continuous variables are presented as median (IQR) and were compared using the Wilcoxon rank-sum test.

**Table 2:** Univariable and multivariable Cox regression analyses for overall survival in the training cohort.

Variable	Category	Univariable HR (95% CI)	*p* value	Multivariable HR (95% CI)	*p* value
NPAR	Per 1-SD increase	2.32 (2.06–2.62)	<0.001	2.19 (1.90–2.53)	<0.001
Age	Per year	1.02 (1.01–1.04)	0.001	1.01 (1.00–1.03)	0.152
Sex		—	0.203	—	0.535
	Male	Reference	—	Reference	—
	Female	0.79 (0.55–1.13)	0.203	0.89 (0.62–1.29)	0.535
Tumor site		—	0.957	—	0.133
	Colon	Reference	—	Reference	—
	Rectum	1.01 (0.72–1.42)	0.957	1.32 (0.92–1.91)	0.133
Pathological stage		—	<0.001	—	<0.001
	I–II	Reference	—	Reference	—
	III–IV	4.18 (2.79–6.25)	<0.001	3.44 (2.27–5.21)	<0.001
CEA	Log10-transformed, per unit	3.31 (2.43–4.52)	<0.001	1.97 (1.42–2.72)	<0.001

The multivariable model included continuous NPAR, age, sex, tumor site, pathological stage, and log10-transformed CEA. NPAR was standardized and analyzed per 1-SD increase. The reference groups were male, colon tumor site, and pathological stage I–II.

**Table 3:** Univariable and multivariable Cox regression analyses for disease-free survival in the training cohort.

Variable	Category	Univariable HR (95% CI)	*p* value	Multivariable HR (95% CI)	*p* value
NPAR	Per 1-SD increase	1.88 (1.66–2.14)	<0.001	1.88 (1.62–2.19)	<0.001
Age	Per year	1.01 (1.00–1.02)	0.129	1.00 (0.99–1.01)	0.884
Sex		—	0.762	—	0.760
	Male	Reference	—	Reference	—
	Female	1.05 (0.76–1.46)	0.762	1.05 (0.75–1.47)	0.760
Tumor site		—	0.746	—	0.542
	Colon	Reference	—	Reference	—
	Rectum	0.95 (0.69–1.31)	0.746	1.11 (0.79–1.55)	0.542
Pathological stage		—	<0.001	—	<0.001
	I–II	Reference	—	Reference	—
	III–IV	2.88 (2.05–4.03)	<0.001	2.65 (1.88–3.75)	<0.001
CEA	Log10-transformed, per unit	2.62 (1.92–3.57)	<0.001	1.75 (1.29–2.37)	<0.001

The multivariable model included continuous NPAR, age, sex, tumor site, pathological stage, and log10-transformed CEA. NPAR was standardized and analyzed per 1-SD increase. The reference groups were male, colon tumor site, and pathological stage I–II.

### 3.2 Association between NPAR and clinicopathological characteristics

**Figure 2:**
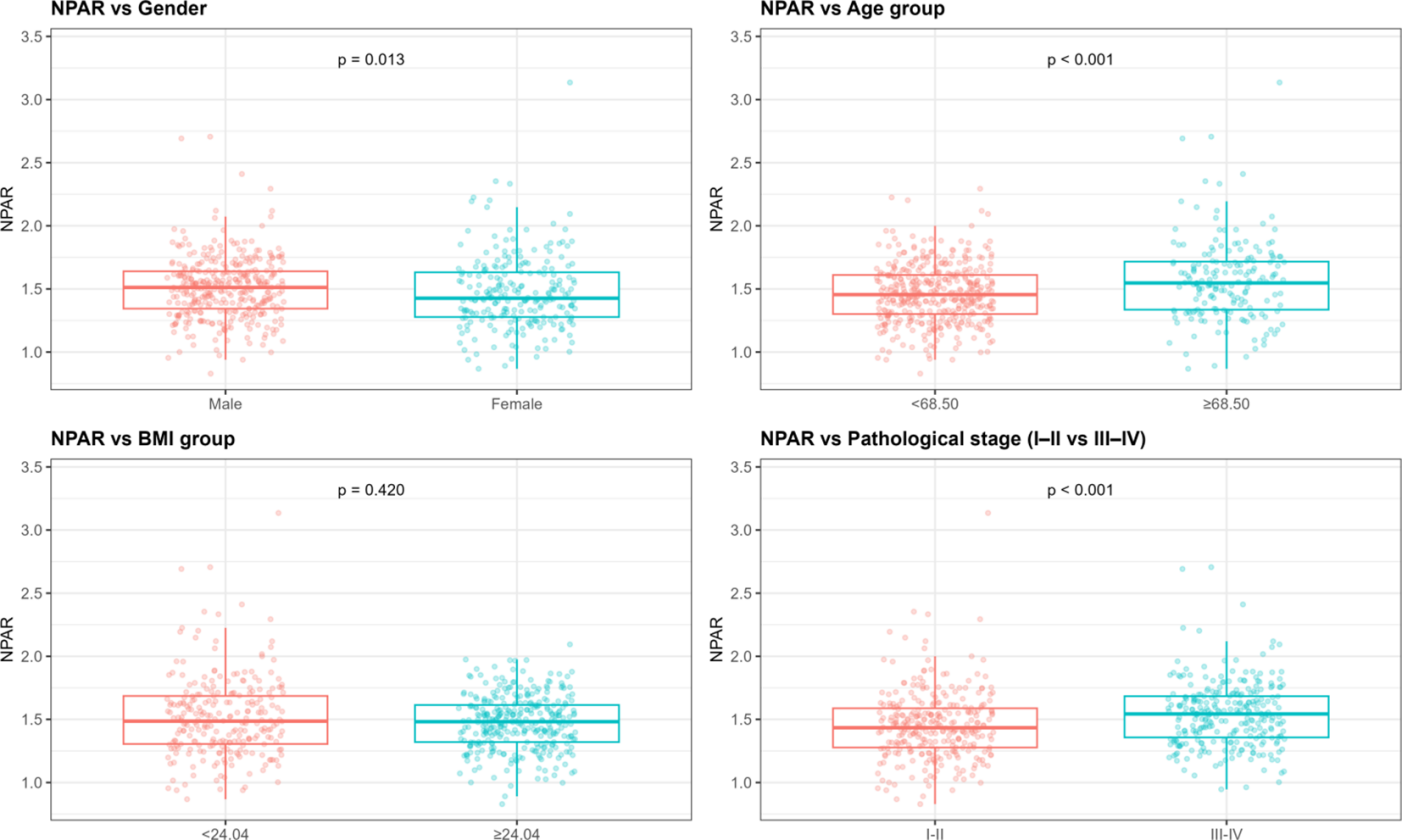
Distribution of NPAR according to sex, age group, BMI group, and pathological stage in the training cohort.

In the training cohort, preoperative NPAR varied with several clinicopathological characteristics (**Figure 2**; **Table 1**). Patients with high NPAR were significantly older than those with low NPAR (age ≥68.5 years: 39.9% vs. 25.4%, *p* < 0.001) and were more likely to be male (66.4% vs. 57.0%, *p* = 0.030). High NPAR was also associated with several adverse tumor characteristics, including more advanced pathological stage (*p* < 0.001), higher frequencies of lymph node metastasis (55.6% vs. 38.8%, *p* < 0.001) and distant metastasis (18.8% vs. 3.4%, *p* < 0.001), larger tumor size (≥4.25 cm: 63.2% vs. 48.6%, *p* < 0.001), and deeper invasion (pT3–4: 84.8% vs. 76.3%, *p* = 0.018). In addition, patients in the high-NPAR group had higher positivity rates for CEA (43.9% vs. 32.4%, *p* = 0.007) and CA125 (9.9% vs. 2.0%, *p* < 0.001). No significant differences were observed in MMR status or CA19–9 status, whereas BMI showed a non-significant trend toward lower values in the high-NPAR group. A dichotomized threshold of 1.55 was identified using the ROC curve and Youden index for OS prediction (AUC 0.85, 95% CI 0.82–0.89, *p* < 0.001; **Supplementary Figure 1**), with a sensitivity of 84% and specificity of 76%; however, because this threshold was data-dependent, continuous NPAR (per 1-SD increment) was used for all primary analyses to avoid optimism. Overall, these associations are consistent with higher NPAR accompanying a more advanced and biologically aggressive disease phenotype, although the magnitude of most differences was modest and there was substantial overlap between groups.

### 3.3 Association of preoperative NPAR with survival outcomes

#### 3.3.1 Association of preoperative NPAR with overall survival

**Figure 3:**
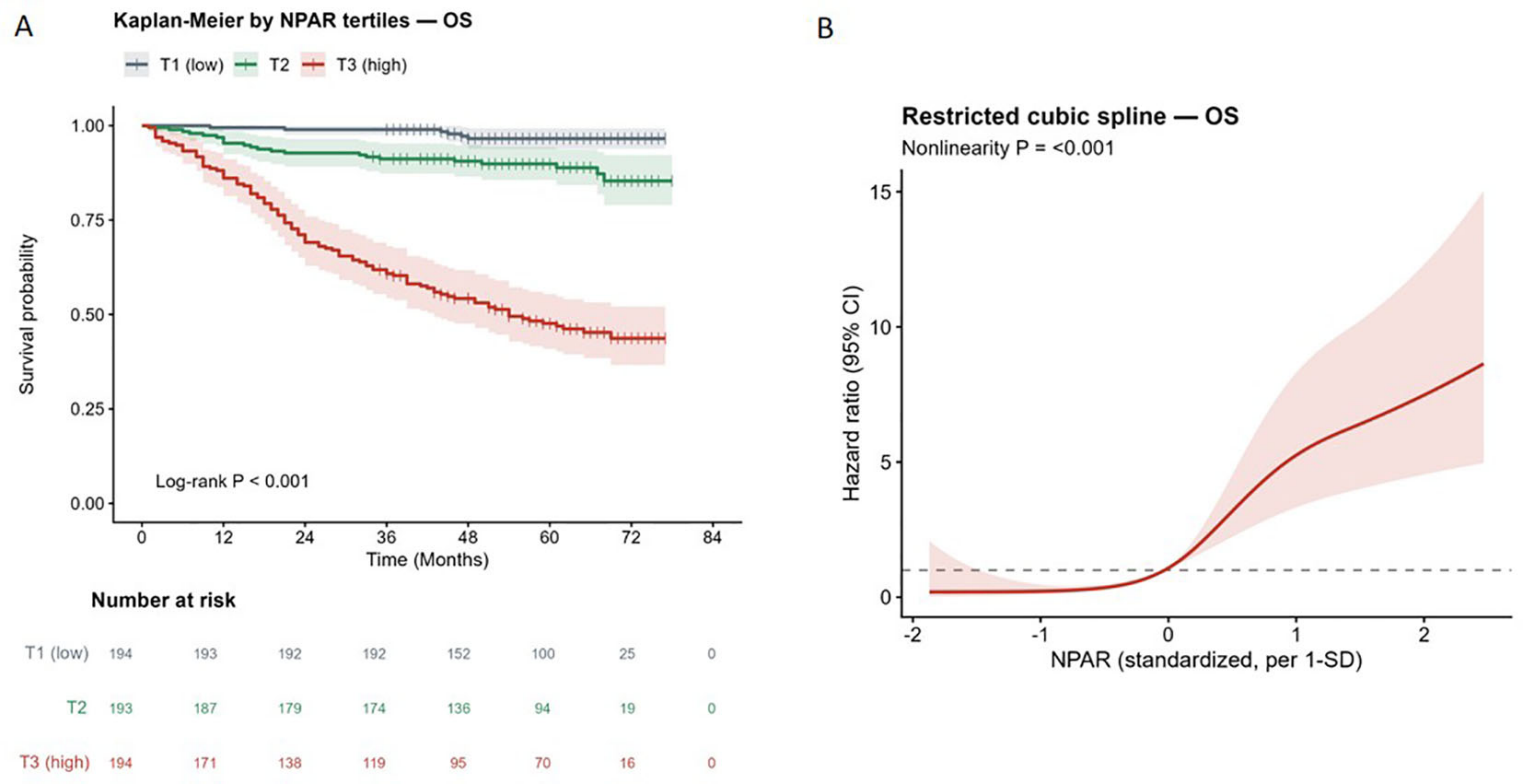
Prognostic value of NPAR for overall survival (OS). **(A)** Kaplan–Meier curves for overall survival stratified by NPAR tertiles. **(B)** Restricted cubic spline showing the association between standardized NPAR and overall survival risk.

When NPAR was analyzed as a continuous variable standardized per 1-SD increase, higher preoperative NPAR was associated with shorter OS in the training cohort. After adjustment for age, sex, tumor site, pathological stage, and log10-transformed CEA, each 1-SD increase in NPAR corresponded to an increased hazard of death (HR 2.19, 95% CI 1.90–2.53, *p* < 0.001; **Table 2**). Kaplan–Meier curves stratified by NPAR tertiles showed a graded separation of OS among the three groups, with patients in higher NPAR tertiles tending to have poorer survival probabilities during follow-up (**Figure 3A**). Restricted cubic spline analysis suggested that the relationship between NPAR and OS was not strictly linear across the full range of NPAR values (*p* for nonlinearity <0.001; **Figure 3B**). The proportional-hazards assumption for NPAR in the OS model was not fully satisfied according to Schoenfeld residual testing (*p* < 0.001; global *p* = 0.003). Therefore, the HR for OS should be interpreted as an average association over the follow-up period rather than as a constant association at all time points. The dichotomized NPAR cutoff of 1.55 was used only for descriptive and supplementary analyses, because this cutoff was derived from the training cohort and may overestimate the effect size when used as the primary exposure definition

#### 3.3.2 Association of preoperative NPAR with disease-free survival

**Figure 4:**
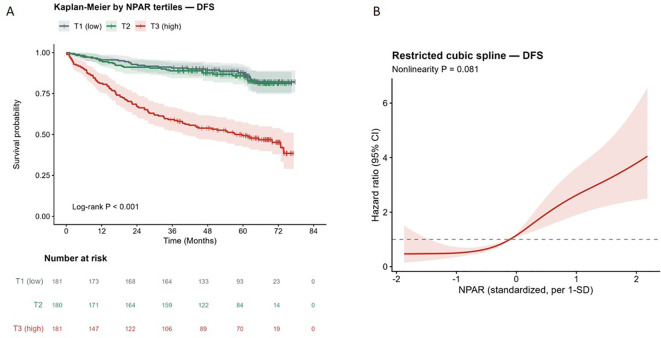
Prognostic value of NPAR for disease-free survival (DFS). **(A)** Kaplan–Meier curves for disease-free survival stratified by NPAR tertiles. **(B)** Restricted cubic spline showing the association between standardized NPAR and disease-free survival risk.

A similar pattern was observed for DFS. In the multivariable Cox model adjusted for age, sex, tumor site, pathological stage, and log10-transformed CEA, each 1-SD increase in NPAR was associated with a higher hazard of DFS events (HR 1.88, 95% CI 1.62–2.19, *p* < 0.001; **Table 3**). Kaplan–Meier curves according to NPAR tertiles showed stepwise separation for DFS, with higher NPAR tertiles tending to have lower disease-free survival probabilities during follow-up (**Figure 4A**). Restricted cubic spline analysis did not show strong evidence of nonlinearity for the association between NPAR and DFS (*p* for nonlinearity = 0.081; **Figure 4B**). Unlike the OS model, the proportional-hazards assumption for NPAR was not rejected in the DFS model based on Schoenfeld residual testing (*p* = 0.149; global *p* = 0.317). These findings support the use of a single summary HR for the association between NPAR and DFS over the observed follow-up period. As with OS, analyses based on the dichotomized NPAR cutoff were presented only as supplementary analyses and were not used as the primary basis for inference.

### 3.4 Development and internal performance of NPAR-based prognostic models

**Figure 5:**
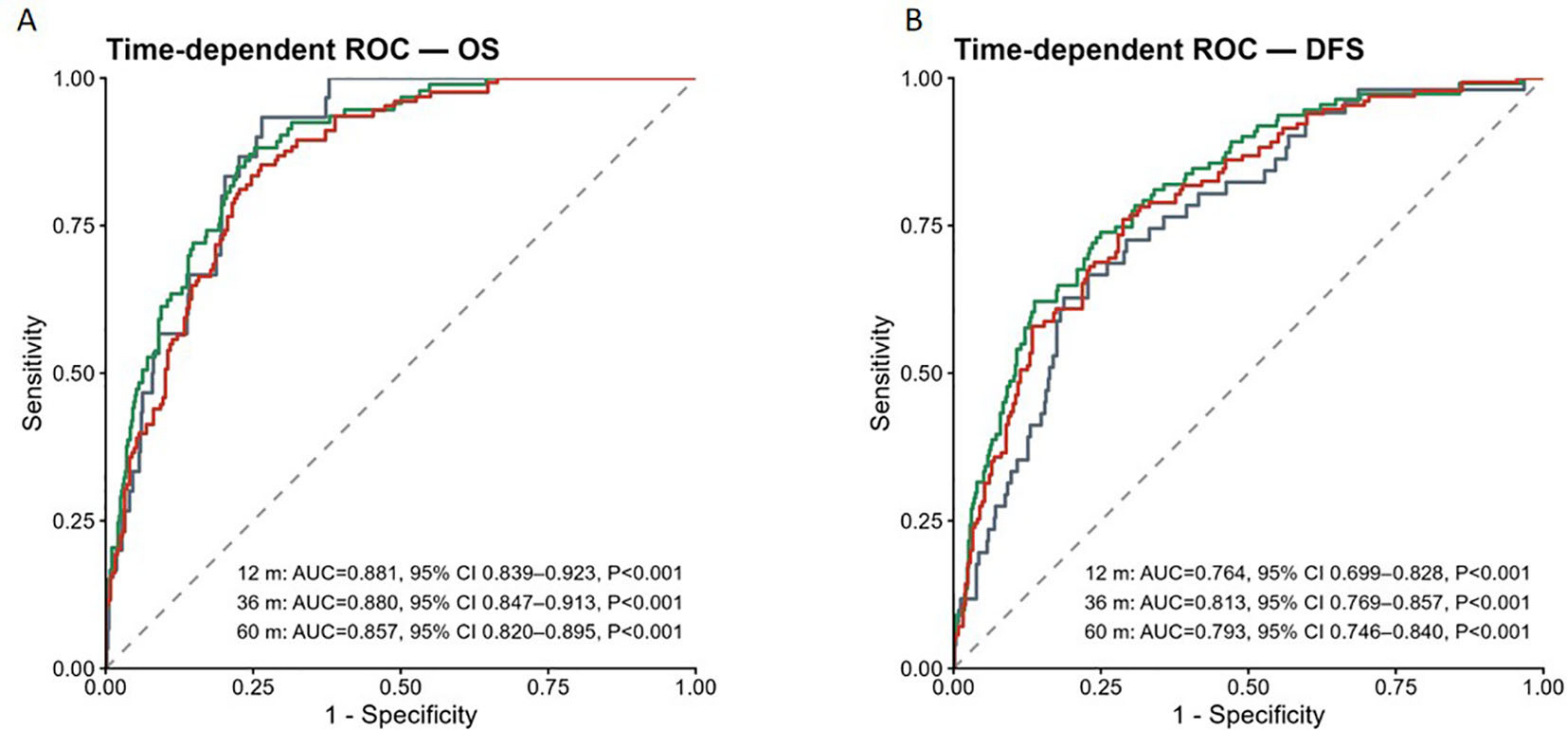
**(A)** Time-dependent ROC curves of the final model for predicting 12-, 36-, and 60-month overall survival in the training cohort. **(B)** Time-dependent ROC curves of the final model for predicting 12-, 36-, and 60-month disease-free survival in the training cohort.

A single pre-specified model was used for both OS and DFS to ensure consistency across model development and performance assessment. The model included continuous NPAR standardized per 1-SD increase, age, sex, tumor site, pathological stage categorized as I–II versus III–IV, and log10-transformed CEA. Individual pT, pN, and pM categories were not entered together with pathological stage, and detailed tumor location was not entered together with tumor site. Collinearity diagnostics did not suggest notable multicollinearity, with variance inflation factors ranging from 1.050 to 1.209 for OS and from 1.057 to 1.159 for DFS. In the training cohort, the model showed reasonable internal performance. The apparent C-index was 0.843 for OS and 0.760 for DFS, and the corresponding optimism-corrected C-indexes were 0.835 and 0.752, respectively. Time-dependent AUCs at 12, 36, and 60 months were 0.881, 0.880, and 0.857 for OS, and 0.764, 0.813, and 0.793 for DFS, respectively (**Figure 5**). Calibration plots showed broadly acceptable agreement between predicted and observed survival probabilities in the training cohort, and decision-curve analysis was used to explore potential clinical utility (**Supplementary Figures 2**–**S4**).

### 3.5 Comparison of baseline characteristics between the training and external validation cohorts

Baseline characteristics available in both the training and external validation cohorts were compared to describe between-cohort comparability before external validation. The results are summarized in **Supplementary Table 1**. The training cohort included 581 patients, whereas the external validation cohort included 483 patients. Overall, the two cohorts showed broadly comparable distributions for sex, pathological stage, baseline NPAR, and CEA. The median NPAR was similar between cohorts, suggesting that the overall distribution of this inflammation–nutrition marker was not substantially different across the two populations. The proportion of patients with stage III–IV disease was also comparable, although some degree of between-center variation was expected because the external cohort was derived from an independent public clinical database. A statistically significant difference was observed in age. Patients in the external validation cohort were slightly younger than those in the training cohort. Although this difference was statistically detectable, the absolute difference in median age was modest. Therefore, it may reflect differences in referral patterns, source populations, or institutional case mix rather than a major clinical imbalance. For continuous variables, including age, NPAR, and CEA, normality was assessed using the Shapiro–Wilk test. Because these variables were not normally distributed, they were summarized as median and interquartile range and compared using the Wilcoxon rank-sum test. Categorical variables were summarized as frequencies and percentages and compared using the chi-square test or Fisher’s exact test, as appropriate. These findings indicate that the external validation cohort was generally suitable for assessing model transportability, while also reflecting real-world between-cohort heterogeneity. Such heterogeneity should be considered when interpreting the attenuation of effect estimates and the lower external model performance observed in subsequent analyses.

### 3.6 External validation of the NPAR-based prognostic models

**Figure 6:**
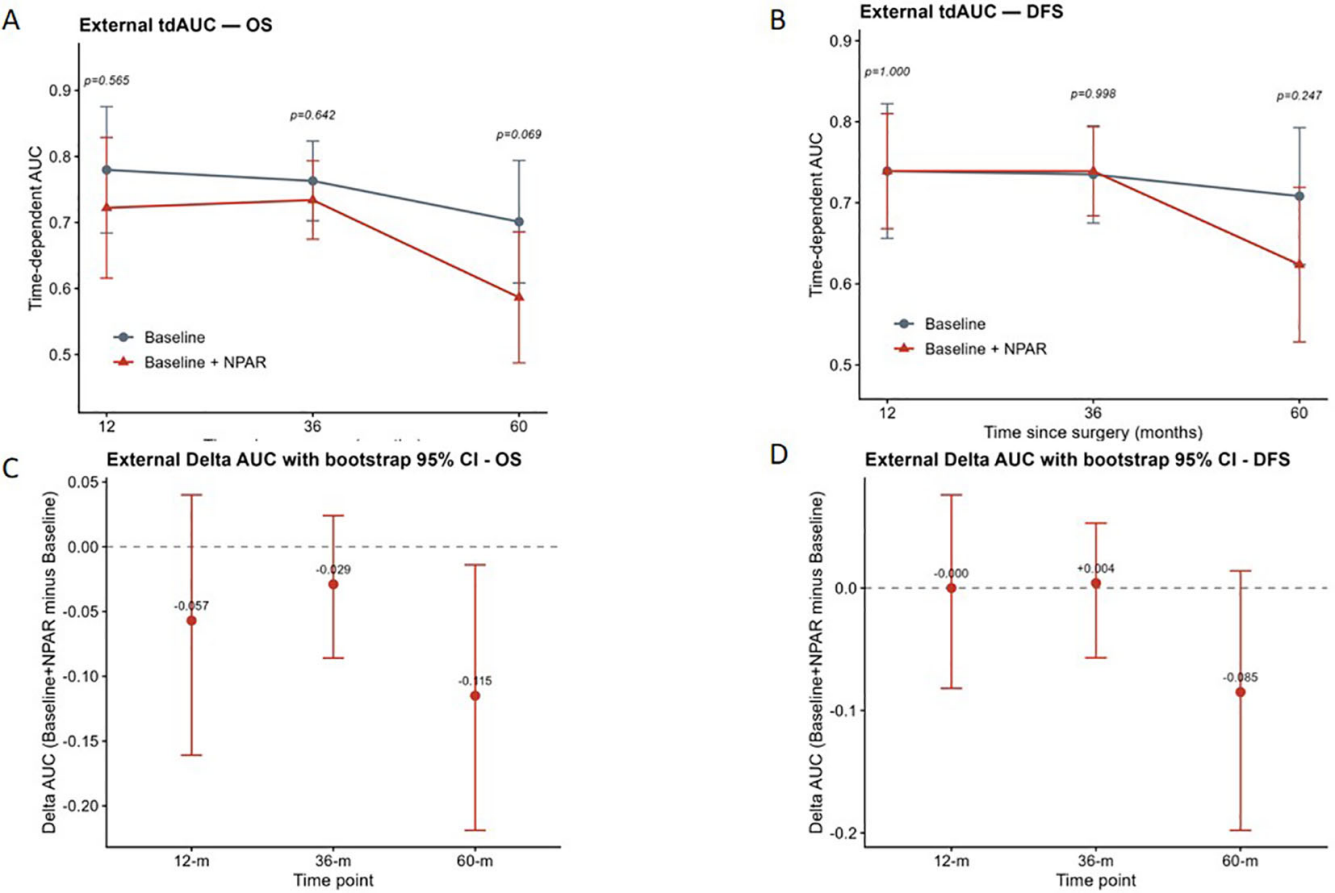
**(A)** External validation of time-dependent AUCs for overall survival comparing the baseline model with the baseline plus NPAR model. **(B)** External validation of time-dependent AUCs for disease-free survival comparing the baseline model with the baseline plus NPAR model. **(C)** Delta AUC with bootstrap 95% confidence intervals for overall survival in the external validation cohort. **(D)** Delta AUC with bootstrap 95% confidence intervals for disease-free survival in the external validation cohort.

External validation was performed in 483 patients from an external clinical database. The model developed in the training cohort was applied to the external cohort without re-estimating the coefficients. When NPAR was analyzed as a continuous variable standardized according to the training-cohort distribution, the direction of association was consistent with that observed in the training cohort, although the magnitude of association was attenuated. Each 1-SD increase in NPAR was associated with OS events in the external cohort (HR 1.21, 95% CI 1.08–1.35, *p* < 0.001) and with DFS events (HR 1.24, 95% CI 1.12–1.37, *p* < 0.001). The external C-indexes of the final model were 0.690 for OS and 0.705 for DFS. The recalibration slopes were 0.370 for OS and 0.489 for DFS, suggesting that the apparent model performance in the training cohort was not fully preserved in the external cohort. These findings indicate limited model transportability and support a cautious interpretation of the training-cohort performance estimates. The incremental contribution of NPAR was evaluated by comparing the final model with a baseline model that included the same clinicopathological variables but excluded NPAR. The external validation results comparing the baseline model with the baseline plus NPAR model are shown in **Figure 6**. For OS, the baseline model showed time-dependent AUCs of 0.780, 0.763, and 0.701 at 12, 36, and 60 months, respectively, whereas the model including NPAR showed AUCs of 0.722, 0.734, and 0.587. The corresponding changes in AUC were −0.057, −0.029, and −0.115. For DFS, the baseline model showed AUCs of 0.739, 0.735, and 0.708 at 12, 36, and 60 months, whereas the model including NPAR showed AUCs of 0.739, 0.739, and 0.624. The corresponding changes in AUC were 0.000, 0.004, and −0.085. Bootstrap confidence intervals for ΔAUC crossed zero at most evaluated time points, except for the 60-month OS estimate, which suggested lower discrimination after adding NPAR. Calibration plots showed some disagreement between predicted and observed survival probabilities in the external cohort, consistent with the low recalibration slopes. Decision-curve analysis did not show a clear and consistent net-benefit advantage of the NPAR-containing model across clinically relevant threshold probabilities (**Supplementary Figures 5**, **S6**). Because CEA was missing in 60 patients in the external cohort, corresponding to 12.4%, multiple imputation using chained equations was performed as a sensitivity analysis. The imputed results were directionally similar to the complete-case analysis, with pooled external C-indexes of 0.691 for OS and 0.695 for DFS, and pooled NPAR HRs of 1.17 for OS and 1.19 for DFS. These findings support the robustness of the association direction but do not materially alter the interpretation that the incremental predictive value of NPAR was limited in the external cohort.

## 4 Discussion

The AJCC 8th edition TNM staging system (6), which serves as the cornerstone of clinical risk stratification in colorectal cancer, is primarily based on tumor morphology. However, it does not account for host-related factors such as systemic inflammation, nutritional status, and immune status, which may limit its ability to explain the prognostic heterogeneity observed among patients within the same stage (21–23). In this study, NPAR was analyzed primarily as a continuous variable standardized per 1-SD increase, and a unified pre-specified final model was applied across Cox regression, nomogram construction, discrimination assessment, calibration, decision-curve analysis, and external validation. Higher preoperative NPAR showed an adjusted association with worse OS and DFS in the training cohort, and the direction of this association was reproduced in the external cohort. However, the magnitude of association was clearly attenuated externally, and adding NPAR did not consistently improve model discrimination, particularly for OS. Therefore, NPAR should be interpreted as an adjunctive inflammation–nutrition-related marker rather than as a biomarker with established incremental predictive value beyond conventional clinicopathological factors.

Existing studies have largely focused on conventional inflammation-based biomarkers, such as the NLR, PLR, and LMR. Although substantial evidence has suggested that these indices are associated with the prognosis of CRC, the reported predictive value has been inconsistent across studies, and nutritional status—an important determinant of patient outcome—has often not been adequately incorporated. By integrating neutrophil percentage and serum albumin concentration, NPAR has been reported as a potentially useful prognostic indicator in several malignancies, including oral cancer and bladder cancer (15, 16). However, evidence regarding its role in colorectal cancer remains limited, particularly with respect to comprehensive prognostic modeling, dynamic performance evaluation, and cross-center validation. All these evaluations were based on the same pre-specified final model, which included continuous NPAR per 1-SD increase, age, sex, tumor site, pathological stage, and log10-transformed CEA. This model consistency allowed the regression results, nomograms, C-index, time-dependent AUC, calibration analysis, decision-curve analysis, and external validation to be interpreted within the same analytical framework.

From a mechanistic perspective, the clinical relevance of NPAR may lie in its ability to simultaneously capture two key dimensions of cancer progression: tumor-promoting inflammation and deteriorating nutritional status. Within the tumor microenvironment, neutrophils exhibit marked biological plasticity and may exert either antitumor or protumor effects. In particular, protumor neutrophil subsets can secrete bioactive mediators such as MMP-9, VEGF, and HGF, thereby promoting angiogenesis, remodeling the extracellular microenvironment, and weakening immune surveillance. In addition, these cells may further aggravate the immunosuppressive microenvironment through membrane expression of PD-L1 and the coordinated release of arginase and nitric oxide, thereby suppressing T-cell activity (24–28). Previous studies have shown that elevated inflammatory indices in patients with CRC are closely associated with reduced OS and DFS, as well as other adverse prognostic features, including poor differentiation and increased CEA levels (29). On the other hand, decreased serum albumin commonly reflects malnutrition, persistent systemic inflammation, or impaired hepatic function, all of which may adversely affect treatment tolerance and overall outcome (30–34). By combining increased neutrophil percentage with decreased albumin, NPAR may provide a more integrated assessment of cancer-related inflammatory burden and host metabolic status. In our study, elevated NPAR was closely associated with advanced tumor stage, larger tumor size, and abnormal tumor marker levels, further supporting its potential role in preoperative risk assessment.

A major methodological issue addressed in this study was the use of dichotomized NPAR. Categorizing a continuous biomarker may lead to information loss, unstable cutoff selection, and overestimation of effect sizes, particularly when the cutoff is derived and tested in the same cohort. Therefore, dichotomized NPAR was retained only for descriptive and supplementary analyses, whereas all primary analyses were based on continuous NPAR. The attenuation of the NPAR effect from the training cohort to the external cohort suggests that the originally large training-cohort effect estimates were likely influenced by optimism and cutoff-related overfitting. Accordingly, the cutoff value of 1.55 should not be regarded as a clinically established threshold, but rather as a study-specific supplementary reference.

From a clinical perspective, NPAR has practical advantages because it is inexpensive, routinely available, and easily calculated from standard preoperative blood tests. However, its clinical role should be interpreted cautiously. Although NPAR may provide information on systemic inflammation and nutritional reserve, the external validation results did not demonstrate a consistent improvement in predictive performance after adding NPAR to conventional clinicopathological variables. Therefore, NPAR should not be regarded as a replacement for TNM staging, CEA, or other established prognostic factors. Instead, it may serve as an adjunctive marker that helps describe the patient’s host-related inflammatory and nutritional status before surgery. Whether this information can improve individualized follow-up planning, perioperative nutritional optimization, or treatment decision-making requires further prospective validation.

The external validation results indicate that the prognostic association of NPAR was reproducible in direction but limited in incremental predictive value. In the external cohort, continuous NPAR remained associated with both OS and DFS, but the effect estimates were substantially smaller than those observed in the training cohort. More importantly, adding NPAR to the baseline clinicopathological model did not improve external discrimination for OS. The time-dependent AUCs of the baseline model were 0.780, 0.763, and 0.701 at 12, 36, and 60 months, respectively, whereas the corresponding AUCs of the NPAR-containing model were 0.722, 0.734, and 0.587. For DFS, the incremental value was also limited, with changes in AUC of 0.000, 0.004, and −0.085 at 12, 36, and 60 months, respectively. These findings suggest that NPAR is associated with outcomes but may not provide stable additional predictive information beyond conventional clinicopathological variables, especially for OS. The discrepancy between internal and external model performance further suggests possible optimism in the training model. Although the optimism-corrected C-indexes in the training cohort were 0.835 for OS and 0.752 for DFS, the corresponding external C-indexes were lower, at 0.690 and 0.705, respectively. In addition, the recalibration slopes were 0.370 for OS and 0.489 for DFS, indicating that predictions derived from the training model were too extreme when applied to the external cohort. These findings support a cautious interpretation of the apparent training-cohort performance and highlight the need for further validation before the model can be used for individualized clinical prediction.

Several limitations of the present study should be acknowledged. First, because this was a retrospective single-center study, selection bias could not be completely avoided, and the findings should therefore be confirmed in future multicenter prospective studies. Second, the external validation cohort was derived from an existing clinical database in which several potentially important variables, including preoperative infection history, chronic inflammatory indicators, and recent use of anti-inflammatory medications, were unavailable. As a result, the exclusion criteria could not be fully matched to those used in the training cohort, which may have affected the reliability of the validation results. Third, key variables such as MSI status, KRAS/BRAF mutations, and postoperative adjuvant therapy were not included in the final model. The absence of adjuvant therapy information is particularly important because postoperative chemotherapy is an important determinant of postoperative OS and DFS outcomes and may act as either a confounder or a mediator in the association between NPAR and survival outcomes. If patients with high NPAR were less likely to receive or complete adjuvant therapy because of poor nutritional status, frailty, systemic inflammation, or postoperative complications, the adverse association between NPAR and survival may have been partially inflated. Conversely, if high-risk patients with elevated NPAR were more likely to receive intensive postoperative treatment, the true adverse prognostic effect of NPAR may have been partially attenuated. Therefore, the reported hazard ratios should be interpreted as associations that may include both biological prognostic effects and treatment-selection effects. Future studies should incorporate adjuvant therapy status, regimen, timing, cycle completion, dose intensity, and treatment-related toxicity. Fourth, owing to the retrospective nature of the data, some potential confounding factors that may influence albumin and neutrophil levels, such as chronic liver disease and occult infection, could not be fully controlled, which may have introduced residual confounding. In the primary analysis, NPAR was modelled as a continuous variable. Restricted cubic spline analysis suggested that the association between NPAR and OS was not strictly linear across the full biomarker range, whereas the association with DFS showed no strong evidence of nonlinearity. In addition, the proportional hazards assumption for NPAR was not fully satisfied in the OS model, indicating that the OS hazard ratio should be interpreted as an average association over follow-up rather than as a constant effect at every time point. Missing data should also be considered when interpreting the findings. In the training cohort, variables included in the final OS model were complete, whereas DFS follow-up information was unavailable in a subset of patients. In the external cohort, CEA was missing in 60 patients, corresponding to 12.4% of the validation sample. Therefore, multiple imputation using chained equations was performed as a sensitivity analysis. The imputed results were directionally similar to the complete-case analysis, with pooled external C-indexes of 0.691 for OS and 0.695 for DFS and pooled NPAR hazard ratios of 1.17 for OS and 1.19 for DFS. These results suggest that missing CEA data did not materially change the main interpretation, although incomplete data remain a source of uncertainty. In summary, preoperative NPAR showed an adjusted association with OS and DFS after adjustment for established clinicopathological variables, and the direction of this association was reproduced in an external cohort. However, the external validation results showed attenuated effect estimates, low recalibration slopes, and limited incremental improvement in discrimination, especially for OS. Therefore, NPAR should be considered a simple adjunctive marker reflecting systemic inflammation and nutritional status, rather than a definitive predictor with established incremental clinical utility. Larger prospective multicenter studies with standardized treatment data and prespecified modeling strategies are required before NPAR can be recommended for routine prognostic decision-making.

## 5 Conclusions

Preoperative NPAR showed an adjusted association with OS and DFS in patients with colorectal cancer undergoing radical surgery. The direction of this association was reproduced in an external cohort. However, the effect estimates were attenuated externally, and adding NPAR to conventional clinicopathological variables did not provide consistent incremental improvement in model discrimination, particularly for OS. Therefore, NPAR should be considered an adjunctive marker for preoperative risk assessment rather than a definitive predictor with established incremental clinical utility. Further large-scale prospective multicenter studies incorporating standardized treatment information, especially postoperative adjuvant therapy, are required to clarify the clinical value of NPAR in prognostic stratification and decision-making.

## Data Availability

The data analyzed in this study is subject to the following licenses/restrictions: The datasets are not publicly available due to restrictions related to patient privacy and ethical approval. Access to the data is limited to protect confidential clinical information and is subject to approval by the corresponding institution and ethics committee. Requests to access these datasets should be directed to Requests to access the datasets should be directed to the corresponding author.
