# Supplementary material for: Association between preoperative neutrophil percentage-to-albumin ratio and survival outcomes in patients with colorectal cancer undergoing radical surgery: a retrospective study with external validation
Source: Front Oncol. 2026 Jul 21;16:1775482. doi: 10.3389/fonc.2026.1775482 (PMC13433297; doi:10.3389/fonc.2026.1775482)
Supplement: Supplementary file 1 [file DataSheet1.docx]

**Supplementary Table S1. Comparison of baseline characteristics between the training cohort and the external validation cohort**

| Variable | Training cohort | External validation cohort | *p* value |
| --- | --- | --- | --- |
| Age | 62.0 (53.00–72.00) | 60.00 (52.00–68.00) | 0.008 |
| Sex |  |  | 0.961 |
| Male | 352 (60.6%) | 291 (60.2%) |  |
| Female | 229 (39.4%) | 192 (39.8%) |  |
| Pathological stage |  |  | 0.808 |
| Ⅰ | 96 (16.5%) | 85 (17.6%) |  |
| Ⅱ | 206 (35.5%) | 178 (36.9%) |  |
| Ⅲ | 225 (38.7%) | 173 (35.8%) |  |
| Ⅳ | 54 (9.3%) | 47 (9.7%) |  |
| NPAR | 1.49 (1.32–1.64) | 1.49 (1.29–1.77) | 0.098 |
| CEA | 3.43 (1.99–8.27) | 3.88 (2.11–8.22) | 0.376 |

**Notes: Continuous variables are presented as median (IQR), and categorical variables as n (%). Age, NPAR, and CEA were assessed for normality using the Shapiro–Wilk test and were found to be non-normally distributed; therefore, between-cohort comparisons were performed using the Wilcoxon rank-sum test. Categorical variables were compared using the chi-square test or Fisher’s exact test, as appropriate. CEA in the external validation cohort was summarized among patients with available CEA measurements.**

**Supplementary Table S2. Summary of missing data in the training and external validation cohorts**

| Variable | Training cohort, missing/total (%) | External validation cohort, missing/total (%) |
| --- | --- | --- |
| Age | 0/581 (0.0%) | 0/483 (0.0%) |
| Sex | 0/581 (0.0%) | 0/483 (0.0%) |
| Tumor site | 0/581 (0.0%) | 0/483 (0.0%) |
| Pathological stage | 0/581 (0.0%) | 0/483 (0.0%) |
| NPAR | 0/581 (0.0%) | 0/483 (0.0%) |
| CEA | 0/581 (0.0%) | 60/483 (12.4%) |
| OS follow-up | 0/581 (0.0%) | 0/483 (0.0%) |
| DFS follow-up | 39/581 (6.7%) | 0/483 (0.0%) |

**Notes: Missingness was summarized for variables included in the final model and for outcome-specific follow-up variables. Outcome variables were not imputed. OS analyses included patients with evaluable OS, whereas DFS analyses included patients with evaluable recurrence or metastasis follow-up. Because CEA was missing in 60 patients in the external validation cohort, multiple imputation by chained equations was performed as a sensitivity analysis.**

**Supplementary Figure S1. ROC analysis for determining the optimal NPAR cutoff value.**

**Supplementary Figure S2. (A) Nomogram for predicting 36- and 60-month overall survival based on the final multivariable model. (B) Nomogram for predicting 36- and 60-month disease-free survival based on the final multivariable model.**

**Supplementary Figure S3. (A) Calibration curve of the final model for 36-month overall survival in the training cohort. (B) Calibration curve of the final model for 60-month overall survival in the training cohort. (C) Calibration curve of the final model for 36-month disease-free survival in the training cohort. (D) Calibration curve of the final model for 60-month disease-free survival in the training cohort.**

**Supplementary Figure S4. (A) Decision curve analysis of the final model for 36-month overall survival in the training cohort. (B) Decision curve analysis of the final model for 36-month disease-free survival in the training cohort.**

**Supplementary Figure S5. (A) Calibration curve of the final model for 36-month overall survival in the external validation cohort. (B) Calibration curve of the final model for 60-month overall survival in the external validation cohort. (C) Calibration curve of the final model for 36-month disease-free survival in the external validation cohort. (D) Calibration curve of the final model for 60-month disease-free survival in the external validation cohort.**

**Supplementary Figure S6. (A) Decision curve analysis of the final model for 36-month overall survival in the external validation cohort. (B) Decision curve analysis of the final model for 36-month disease-free survival in the external validation cohort.**
